# Diagnostic Disagreements in Bipolar Disorder: The Role of Substance Abuse Comorbidities

**DOI:** 10.1155/2012/435486

**Published:** 2012-01-26

**Authors:** Rowena Shalini Theodore, Monica Ramirez Basco, John R. Biggan

**Affiliations:** Department of Psychology, University of Texas at Arlington, P.O. Box 19528, Arlington, TX 76019, USA

## Abstract

Substance abuse can produce symptoms similar to other psychiatric disorders, thus confusing the diagnostic picture. This paper attempts to elucidate how misdiagnosis in bipolar disorder might be explained by the presence of substance abuse comorbidities. The overlap of symptoms, limited information about symptom onset, and inexperienced clinicians can result in the misinterpretation of symptoms of substance abuse disorders for bipolar disorder. The present study found that the presence of a substance abuse comorbidity, the polarity of last episode (depressed, manic, mixed, not otherwise specified), and the total number of comorbidities affected the reliability of a bipolar disorder diagnosis.

## 1. Introduction

Clinically, the symptoms of Bipolar Disorder (BPD) during manic episodes are quite distinct and relatively easy to identify including elevated mood, rapid speech, agitation, and participation in high-risk behaviors [1]. However, during depressive, mixed, or hypomanic episodes, or when accompanied by psychotic features, BPD shares symptoms with major depressive disorder, schizophrenia, substance abuse disorders, and several personality disorders and can therefore be difficult to distinguish.

It is this overlap in symptoms that makes the diagnostic process challenging [2–4]. In fact, misdiagnosis is common in BPD [5, 6]. For example, Zimmerman and colleagues [6] examined 700 psychiatric patients who reported that they had been previously diagnosed with BPD. Each person was reevaluated using the Structured Clinical Interview for DSM Disorders (SCID for DSM-IV) [7]. They found that only 43.4% of patients who claimed they had been previously diagnosed with BPD met criteria based on the SCID. This is consistent with other studies [5, 8].

The consequences of an incorrect diagnosis are apparent. Treatment decisions are based on diagnosis and, therefore, inadequate and/or incorrect pharmacological treatments might be applied which lead to unpleasant side effects without the benefit of symptom reduction [9]. These consequences are costly with regard to human suffering and health care service utilization [3].

 In addition to overlapping symptoms, comorbidities such as substance abuse, which occurs in 65% of those diagnosed with BPD [10], can produce symptoms that muddle the diagnostic picture [11]. Goldberg and colleagues [5] interviewed patients with substance abuse problems using structured diagnostic interviews during substance-free time periods. They found that only 32.9% of participants previously diagnosed with BPD met full DSM-IV criteria for bipolar I or II disorders. This suggests that prior substance use had contributed to the misdiagnosis. Likewise, Stewart and El-Mallakh [12] studied patients in a substance-abuse treatment program who had been previously diagnosed with BPD. They found that only 42.9% of participants met criteria for BPD.

Substance abuse disorders are prevalent comorbidities among people with BPD [13]. These disorders may begin as primary disorders or may result from self-medication to reduce or alleviate symptoms of BPD [14]. Commonly abused substances include alcohol, cannabis, cocaine, and stimulants. Not all clinicians are familiar with the signs and symptoms of substance abuse and dependence and could easily mistake them as evidence of a mood disorder [15] because of their effect on mood and behavior. Substance intoxication or withdrawal symptoms may present as symptoms of mania or depression, respectively, thereby misleading clinicians [16]. Errors can easily occur if clinicians rely too much on global heuristics to diagnose patients rather than thoroughly evaluating all symptoms of a disorder [17, 18].

Studies have shown that utilizing structured diagnostic assessments can improve diagnostic accuracy across psychiatric disorders (e.g., [8, 19]), but less specific guidance has been provided regarding the mistakes made by diagnosticians and how they might be avoided. A better understanding of common sources of error in diagnosis might provide clinicians who do not have access to structured diagnostic methods, such as the SCID, with information that improves the accuracy of their diagnoses. For example, if prior or concurrent substance abuse or dependence is common among patients about whom clinicians disagree on a diagnosis of BPD, then comorbid mood symptoms and substance use might cue the need to invest more time and effort in gathering diagnostic information. Similarly, since structured diagnostic assessments are time intensive and costly, they cannot be provided for all patients. If it was determined that diagnostic error was more likely to occur for those suspected of having BPD along with several comorbidities, then using structured methods might be justified in these cases.

 The present study reexamined diagnostic accuracy data from Basco et al., [8] to determine if cases in which clinicians disagreed on a diagnosis of BPD could be explained by the presence of substance abuse or dependence, number of comorbidities, or polarity of last episode. Disagreements were cases in which a primary diagnosis of BPD was given by either a treating psychiatrist using routine clinical methods, a nurse using the SCID, or an expert diagnostician using all available data, but was not confirmed by the other sources. It was hypothesized that the presence of substance use disorders would lead to greater diagnostic disagreement because these disorders would present with mood symptoms that could be misinterpreted as a mood disorder. Additionally, the total number of comorbidities identified by the expert or gold standard diagnostician was compared for cases in which diagnostic agreement was achieved between clinicians as compared to those in which there was disagreement. It was hypothesized that a greater number of comorbidities occurring concurrently with BPD would be consistent with more diagnostic discrepancies. Finally, the polarity of the most recent episode was evaluated to determine if discrepancies were more likely to occur when the patient was in a manic, depressed, or mixed state. It was hypothesized that there would be fewer diagnostic discrepancies when patients presented with manic symptoms than with mixed or depressive symptoms as manic symptoms tended to be more striking and stereotypic of the disorder.

## 2. Method

### 2.1. Sample

Participants were recruited through clinician referrals and advertisements offering free diagnostic evaluation in a community mental health center. Only participants from the Basco et al. [8] sample who had been diagnosed with BPD by the clinic psychiatrist, study nurse (with or without medical records), or by the expert gold standard diagnosticians, were included in sample. This resulted in a subsample of 120 patients aged 19 to 65 who were primarily female and Caucasian (see Table 1). All were economically disadvantaged and treated in a clinic for the care of persons with severe mental illnesses. The Institutional Review Board at the University of Texas Southwestern Medical Center at Dallas approved the study, and all participants signed an informed consent to participate. Participants were compensated $20.00 for completion of diagnostic and follow-up interviews. Gold standard diagnoses were explained to patients by the expert diagnostician, and they were provided with the opportunity to relay the diagnostic information to their treating physician by signing a release of records form. 

### 2.2. Procedure

At the time of his or her initial intake evaluation at the clinic, each patient was interviewed by a clinic psychiatrist without the use of structured diagnostic instruments per routine clinic procedures (routine diagnosis). At study entry, participants underwent a Structured Clinical Interview for DSM-IIIR (SCID) administered by a trained psychiatric nurse. During the diagnostic interview, the general medical history of each patient was recorded, as well as his or her family history of mental disorders. Life charts [20] representing a timeline of the patients' symptoms, including substance use behaviors, were also constructed. Following the SCID, the study nurse documented the diagnosis derived from the SCID interview (SCID diagnosis).

The nurse reviewed each patient's medical records and the SCID diagnosis was updated (SCID + medical records diagnosis) if the additional information suggested a different diagnosis. Finally, an expert doctoral level diagnostician reviewed the SCID, medical history, family mental health history, and life charts and conducted a follow-up interview with each patient to explore differential diagnoses and to rule out or confirm the SCID + medical records diagnoses (gold standard diagnosis). For the present study, the routine, SCID, and SCID + medical records diagnoses were combined as a “pregold standard” diagnosis.

Because the gold standard diagnoses were assumed to be most accurate, they were used to group patients as having either “alcohol abuse or dependence,” “drug abuse or dependence,” “alcohol and drug abuse or dependence,” or “neither alcohol nor drug abuse or dependence.” In addition, the total number of diagnoses rendered by the “gold standard” for each patient was recorded. In this sample, comorbidities included substance or alcohol use disorders, panic disorder (with and without agoraphobia), obsessive-compulsive disorder (OCD), generalized anxiety disorder (GAD), and posttraumatic stress disorder (PTSD).

## 3. Results

Diagnostic agreement and disagreement between the “pre-gold standard” and the “gold standard” evaluation was analyzed. Chi-square tests of independence were used for all tests below. The expected values for the chi-square tests were obtained by averaging the proportion of agreements and disagreements between the groups and multiplying this average by each of the total number of observations for each group. This was done to reduce the influence of disproportionate cell sizes.

### 3.1. Substance Use Disorders

**Figure 1:**
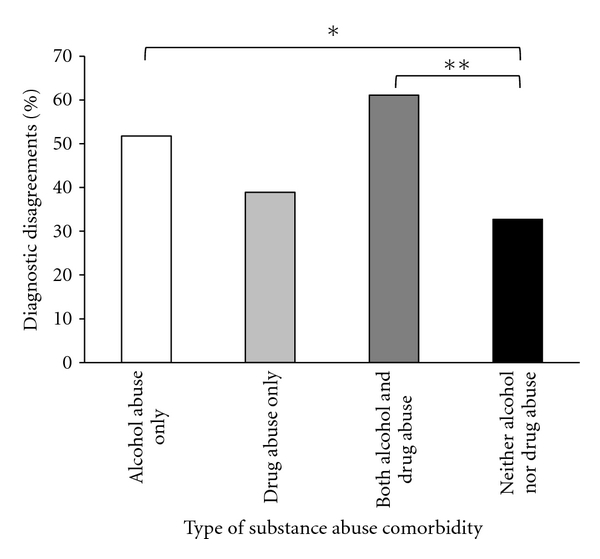
Percentage of diagnostic disagreements between the “pregold standard” diagnosis and the “gold standard” diagnosis by the type of substance abuse that was present. *P < 0.10, **P < 0.05.

The effect of the presence or absence of a substance use disorder on diagnostic agreement and disagreement was assessed using a chi-square test with four categories: alcohol only, drugs only, both alcohol and drugs, and no substance use disorder (Figure 1). The omnibus test found marginally significant differences in the amount of diagnostic agreement and disagreement between the four groups, *χ* ^2^ (3, *N* = 120) = 6.34, *P* = 0.096 (see Table 2).

A post hoc analysis revealed that there were marginally more diagnostic disagreements among patients in the alcohol only group (51.7%) relative to the no substance use disorder group (32.7%), *χ* ^2^ (1, *N* = 84) = 3.11, *P* = 0.078. There were also more diagnostic disagreements among the both alcohol and drug group (61.1%) than the no substance use group (32.7%), *χ* ^2^ (1, *N* = 73) = 5.90, *P* < 0.05. A comparison of diagnostic disagreements between the both alcohol and drug group (61.1%) and the drug only group (38.9%) showed no statistical differences. 

### 3.2. Number of Comorbidities

**Figure 2:**
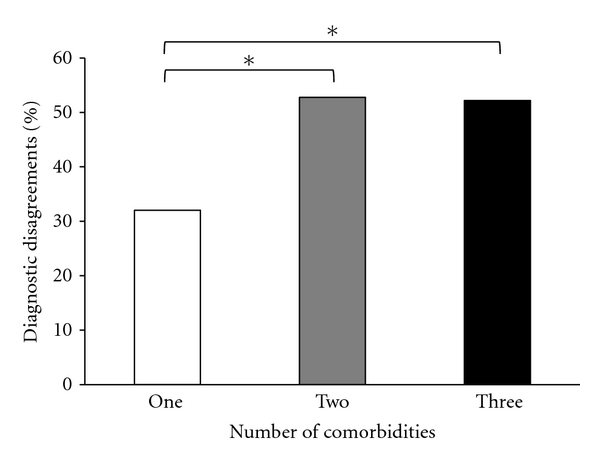
Percentage of diagnostic disagreements between the “pre-gold standard” diagnosis and the “gold standard” diagnosis by the total number of comorbidities. *P < 0.10.

To test the hypothesis that an increase in number of comorbidities would be related to diagnostic disagreements, the numbers of comorbidities were treated as groups and evaluated using a chi-square test. Cochran [21] stated that chi-square tests become unreliable when 20% of cells contain values that are less than five. Therefore, participants with more than three comorbidities were removed as the cell sizes were small and the expected values for each of these groups (four = 4.05, five = 0.45, and six = 0.45) were less than five. Therefore, the number of diagnostic disagreements was compared for patients with one, two, and three comorbidities (Figure 2). The number of diagnostic disagreements showed marginally significant differences between the groups, *χ* ^2^ (2, *N* = 109) = 4.89, *P* = 0.087 (see Table 4).

Post hoc comparisons determined that there were marginally more diagnostic disagreements for patients with either two (52.8%), *χ* ^2^ (1, *N* = 73) = 3.05, *P* = 0.051, or three (52.1%) comorbidities, *χ* ^2^ (1, *N* = 73) = 3.05, *P* = 0.081 than only one (32%) comorbidity. There was no significant difference in the number of diagnostic disagreements for patients with two (52.8%) compared with three (52.1%) comorbidities.

### 3.3. Polarity of Most Recent Episode

**Figure 3:**
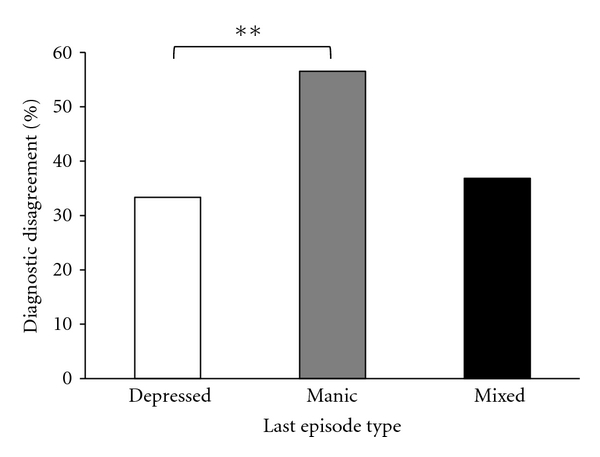
Percentage of diagnostic disagreements between the “pre-gold standard” diagnosis and the “gold standard” diagnosis by the type of the last episode experienced. **P < 0.05.

For the comparison of agreements and disagreements by the type of the last episode, patients whose last episode was Not Otherwise Specified (NOS) were removed. An omnibus test of the influence of the type of the most recent episode (Depressed, Manic, or Mixed) on diagnostic disagreement revealed no significant differences between the groups (see Table 3). However, a planned post hoc comparison of the number of diagnostic disagreements for those patients whose most recent episode was Depressed (33%) with patients whose most recent episode was Manic (57%) found that significantly more diagnostic disagreements occurred for patients whose most recent episode was Manic, *χ* ^2^ (1, *N* = 77) = 4.18, *P* < 0.05 (see Figure 3). None of the other comparisons showed statistically significant differences.

## 4. Discussion

The process of assessing psychiatric diagnoses, which relies on patient self-report of symptoms, clinical judgment, experience, and intuition to some extent, is quite complicated. Most studies of the accuracy of diagnosis [3, 6] attest to this. Structured methods have improved the process [8, 19], but because of the sole reliance on clinical observation and decision-making in the absence of available precise laboratory measures, even these methods are subject to error. The purpose of this research was to attempt to identify clinical features that might explain discrepancies in diagnoses among clinicians, thus providing indicators for heightened sensitivity to diagnostic complexity and the potential for error. Specifically, given the high prevalence of substance use disorders among those with BPD, the association between diagnostic disagreements and the presence of substance use disorders was evaluated. Consistent with previous studies [5, 12], we found that there were, in fact, more diagnostic disagreements for patients diagnosed with BPD who met criteria for comorbid alcohol or substance abuse or dependence (alcohol only or both alcohol and drugs) than for those who did not have substance use disorders. These discrepancies may be due, in part, to the fact that substance intoxication can affect mood (i.e., induce euphoria), disrupt cognitive functioning, and lead to risk taking behaviors, all of which are common in BPD. Withdrawal can be mistaken for symptoms of depression such as dysphoric mood, lethargy, and sleep disturbance. Intoxication and withdrawal can also produce psychotic symptoms such as hallucinations [2], which are not uncommon in the depressive and manic phases of BPD.

It appears that the presence of one co-morbid psychiatric disorder did not cloud the diagnostic picture, as it was not associated with diagnostic disagreement. Clinicians could differentiate one additional disorder from the primary mood disorder. However, the presence of more than one comorbid disorder appeared to contribute to diagnostic confusion, most likely due to the overlap in symptoms among disorders. Similarly, Zimmerman and colleagues [11] found that the errors in the diagnosis of BPD occurred more frequently in patients with three or more comorbid disorders.

 The patients in this sample had comorbidities other than substance use disorders, with anxiety disorders being the second most common. It is not unusual for people with BPD to also experience considerable anxiety that presents in various forms [10]. Likewise, anxiety symptoms such as irritability and psychomotor agitation can be present in both the depressive and manic phases of BPD. While we did not examine the relationship between specific anxiety disorders and BPD diagnostic agreement, our findings on number of comorbidities may suggest that additional caution be exercised during the diagnostic process when anxiety and BPD symptoms are present.

Past research [22, 23] has shown that substance abuse is associated with an increased likelihood of transition in episode polarity, a transition during which a mix of depressive and manic symptoms can confuse the diagnostic picture. In addition, patients who have more than one comorbid substance abuse disorder may have a greater variety of mood fluctuations, thus creating a complex picture that is difficult to disentangle without the opportunity to observe the patients during periods of abstinence.

With limited time and without extensive diagnostic and historical information, clinicians are often forced to rely on decision-making heuristics [17, 18] as they attempt to produce the most accurate diagnosis. Unfortunately, the use of heuristics to form a diagnosis can lead to significant misjudgments as it relies heavily on personal preconceptions and past experiences, which are influenced by selective memory and clinical experience that varies greatly across clinicians and over time [3]. For example, perhaps a depression heuristic is activated when clinicians observe symptoms of major depression that are somewhat different from the typical presentation of patients with a unipolar mood disorder. In our sample, patients with a most recent episode of major depression were more likely to be accurately diagnosed with BPD compared to those with a most recent episode of mania. However, overreliance on this heuristic could also lead to inaccuracies as suggested by the finding that patients who were abusing a depressant (i.e., alcohol) were more likely to be incorrectly diagnosed with BPD.

### 4.1. Limitations

A major limitation of this research was its sample size given that it was derived from a previous study [8]. This limitation precluded investigation of other factors, such as demographics, that might have been associated with greater diagnostic disagreement. The thrust of the Basco et al. [8] study, from which these data were derived, was that the greater the amount of information available, the more accurate clinicians tended to be in their evaluations. Age, for example, can be a proxy for length of illness, with older patients potentially having had more episodes and therefore providing more diagnostic data for their life charts to help distinguish substance-induced mood episodes from co-morbid BPD and substance use disorders. In our sample, there was significantly greater diagnostic disagreement (66.7%) for patients under the age of 30 than for older patients (37.9%), **χ** ^2^ (1, *N* = 77) = 10.06, *P* < 0.01. This may be an artifact of providing more information from which to derive a diagnosis.

This study was also based on patients in a single mental health center which may limit the generalizability of the findings. Furthermore, due to the small sample size, this study focused on diagnostic disagreements among clinicians, not on the accuracy of the initial diagnosis (i.e., also comparing patients who were not diagnosed with BPD who, in fact, had BPD). Thus, we are unable to conclude that the presence of substance abuse comorbidities increased the likelihood of a patient with BPD not being diagnosed with BPD. Replication of this study in different clinical settings with a larger sample size would help address these issues.

Additionally, the diagnoses in this study were made using the DSM-III-R. This may raise a concern that these findings would be different if criteria from the DSM-IV-TR were used instead. However, a comparison of the diagnostic criteria for BPD in the DSM-III-R and the DSM-IV-TR found no significant differences. It is, therefore, believed that the results of this study are applicable to the diagnostic procedures of the DSM-IV-TR.

For future research and elaborations on this study, it would be of interest to study the effects of individual substances on the diagnostic accuracy of BPD. Due to sample size restrictions, comparisons were limited to a combined level. However, if research is able to determine that the consumption of a specific drug affects the diagnostic reliability of a BPD diagnosis, this will enable clinicians to identify the exact category of drug that causes symptom confusion, which will help to simplify diagnostic procedures.

## 5. Conclusions

This study attempted to explain some of the factors that might interfere with diagnostic accuracy in a community mental health sample of patients with significant mood symptoms. It was found that the presence of substance abuse or dependence, symptoms of mania, and increased number of comorbidities were related to diagnostic disagreements for bipolar disorder. These findings are not surprising. Experienced diagnosticians can attest to the fact that the more complicated the symptom presentation, the more difficult it is to accurately disentangle symptoms, particularly when the same symptoms are common across several disorders.

The draw of substance use is often the alteration in mood. It is this effect that contributes to the confusion in psychiatric evaluations. While structured methods can help organize diagnostic information, clinicians must still make judgments as to the origin of symptoms (i.e., substance related or not). What our findings suggest is that when manic symptoms are present and a substance use history is endorsed, extra caution should be taken in compiling a detailed history of the onset and offset of each. If substance use predates symptom onset that is close in time, a substance-related mood disorder diagnosis is likely. If self-medication with substances of abuse occurs after the onset of mood symptoms, then a mood disorder may be more likely. Comorbidities are best sorted out by use of a life chart [20] or time line where the onset and offset of BPD symptoms and substance abuse symptoms can be documented. This method was used in the original study to help differential diagnoses. However, when mood and substance use symptoms occur simultaneously, it may not be possible to differentiate the two until the patient discontinues his or her use of substances long enough for its effect on symptoms to dissipate.

As an additional note, as the mental health community prepares for the introduction of the DSM-V (expected in 2013), this is an opportunity to make adjustments to highlight the importance of ruling out substance abuse disorders when diagnosing a patient with BPD, as well as to clarify the differences between both disorders. 

## Figures and Tables

**Table 1:** Participant demographic characteristics.

Characteristic	*n*
Gender	
Female	78
Male	43
Ethnicity	
White	92
Hispanic	10
African American	18
Native American	1
Marital Status	
Single, never married	27
Married	26
Divorced	41
Other	27
Education	
Post-high school	43
High-school	78

**Table 2:** Frequencies of substance abuse disorders.

Categories	*n* (120)	Disagreements
Alcohol abuse	29	15 (51.7%)
Alcohol abuse	4	
Alcohol dependence	25	
Drug abuse only	18	7 (38.9%)
Amphetamine dependence	2	
Cannabis abuse or intoxication	3	
Cannabis dependence	2	
Cocaine abuse or intoxication	1	
Cocaine dependence	1	
>1 type of drug abuse	10	
Both alcohol and drug abuse	18	11 (61.1%)
Neither alcohol nor drug abuse	55	18 (32.7%)

**Table 3:** Frequencies of last episode type.

Categories	*n* (120)	Disagreements
Depressed	54	18 (33.3%)
Manic	23	13 (56.5%)
Mixed	19	7 (36.8%)
NOS	24	13 (54.2%)

**Table 4:** Frequencies of number of comorbid diagnoses.

Categories	*n* (120)	Disagreements
One	50	16 (32.0%)
Two	36	19 (52.8%)
Three	23	12 (52.2%)
Four	9	3 (33.3%)
Five	1	1 (100%)
Six	1	0 (0.00%)
